# AOH1996 targets mitochondrial dynamics and metabolism in leukemic stem cells via mitochondrial PCNA inhibition

**DOI:** 10.1186/s40164-024-00586-4

**Published:** 2024-12-28

**Authors:** HyunJun Kang, Melissa Valerio, Jia Feng, Long Gu, Dinh Hoa Hoang, Amanda Blackmon, Shawn Sharkas, Khyatiben Pathak, Jennifer Jossart, Zhuo Li, Hongyu Zhang, Bin Zhang, Patrick Pirrotte, J. Jefferson P. Perry, Robert J. Hickey, Linda Malkas, Guido Marcucci, Le Xuan Truong Nguyen

**Affiliations:** 1 Department of Hematologic Malignancies Translational Science, Beckman Research Institute and City of Hope National Medical Center, Duarte, CA USA; 2 Department of Hematology, Peking University Shenzhen Hospital, Shenzhen, China; 3 Department of Molecular Diagnostics & Experimental Therapeutics, Beckman Research Institute of City of Hope, Duarte, CA USA; 4 Early Detection and Prevention Division, Translational Genomics Research Institute, Phoenix, AZ USA; 5 Beckman Research Institute, City of Hope National Medical Center, Duarte, CA USA; 6 Department of Molecular Medicine, City of Hope National Medical Center, Duarte, CA USA; 7 City of Hope, Duarte, CA 91010 USA

**Keywords:** AOH1996, PCNA inhibitor, Mitochondrial metabolism, Leukemic stem cells, AML

## Abstract

Cytoplasmic proliferating cell nuclear antigen (PCNA) is highly expressed in acute myeloid leukemia (AML) cells, supporting oxidative metabolism and leukemia stem cell (LSC) growth. We report on AOH1996 (AOH), an oral compound targeting cancer-associated PCNA, which shows significant antileukemic activity. AOH inhibited growth in AML cell lines and primary CD34 + CD38 − blasts (LSC-enriched) in vitro while sparing normal hematopoietic stem cells (HSCs). In vivo, AOH-treated mice demonstrated prolonged survival compared to controls (50 vs. 35 days; *p* < 0.0001) with reduced LSC burden, as shown in secondary transplants (42 vs. 30 days, *p* < 0.0001). Mechanistically, AOH disrupted mitochondrial PCNA’s binding to the OPA1 protein, enhancing OPA1’s interaction with its E3 ligase, MARCH5, which led to OPA1 degradation. This process reduced mitochondrial length, fatty acid oxidation (FAO), and oxidative phosphorylation (OXPHOS), thereby inhibiting LSC expansion. The addition of venetoclax (VEN), an FDA-approved Bcl-2 inhibitor, further enhanced AOH’s effects, reducing mitochondrial length, FAO, and OXPHOS while improving survival in AML models. While VEN is approved for AML, AOH is under clinical investigation for solid tumors, and our findings support its broader therapeutic potential.

**Supplementary Information:**

The online version contains supplementary material available at 10.1186/s40164-024-00586-4.

## To the Editor,

Proliferating cell nuclear antigen (PCNA) is involved in tumor DNA synthesis and repair, and disease progression [1, 2]. In contrast to its nuclear role, cytoplasmic PCNA maintains mitochondrial DNA integrity, regulates mitochondrial dynamics (such as fission and fusion), and mediates cellular stress responses by supporting mitochondrial function under stress conditions [3, 4]. In acute myeloid leukemia (AML), cytoplasmic PCNA is highly expressed, supporting oxidative metabolism and growth, especially in leukemia stem cells (LSCs) [5]. LSCs reportedly rely on mitochondrial fusion, fatty acid oxidation (FAO), and oxidative phosphorylation (OXPHOS) for survival [6, 7]. Therefore, targeting cytoplasmic PCNA could disrupt LSC homeostasis, leading to their elimination and potential disease eradication. Building on the previous PCNA inhibitor AOH1160, we developed a leading clinical candidate AOH1996, which is orally administrable and metabolically stable and showed significant inhibition of tumor growth with minimal toxicity for healthy cells [8, 9].

**Fig. 1:**
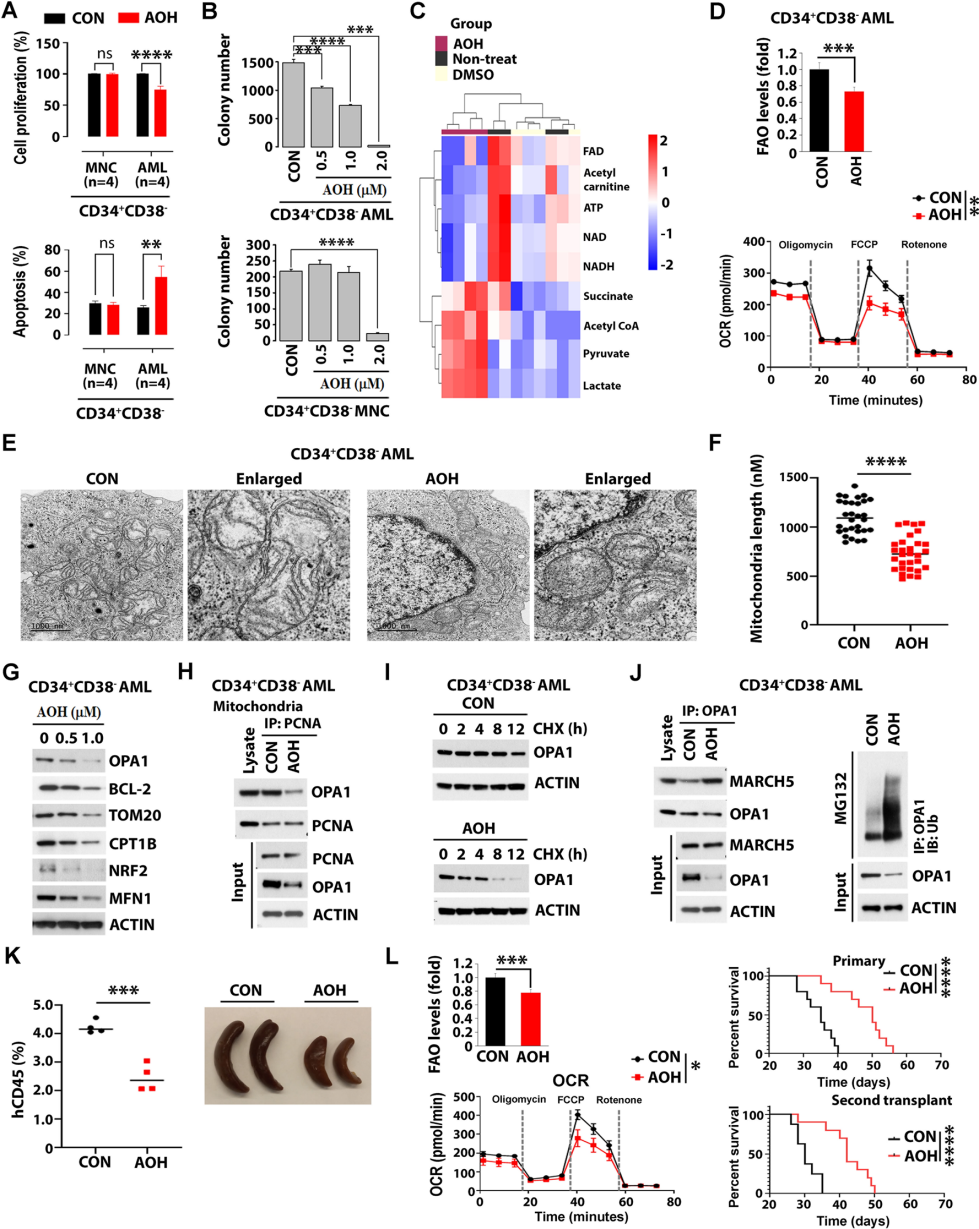
Impact of AOH1996 (AOH) on leukemic stem cells and leukemogenesis via inhibition of mitofusion and mitochondrial metabolism. **A** Effects of AOH (1 µM) on proliferation and apoptosis of LSC-enriched AML blasts. CD34 + CD38- cells were isolated from primary MNCs (*n* = 4) or AML blasts (*n* = 4). Top, cell proliferation levels. Bottom, apoptosis levels. **B** Effects of AOH on colony formation of LSC-enriched AML blasts. CD34 + CD38- AML blasts (top) or MNCs (bottom) (2 × 10⁵ cells/mL, *n* = 3) were treated with DMSO control (CON) or indicated doses of AOH for 24 h, then plated in methylcellulose. After 14 days, colonies were imaged using a light microscope and counted. Data are shown as mean ± SE, with triplicate determinations. **C** Unsupervised hierarchical clustering of significantly different (adj. *p* < 0.05) metabolites from primary CD34 + AML blasts treated with AOH (0.5 µM), DMSO or Non-treat controls for 24 h. Metabolite changes were displayed as a heat map. **D-F** Primary CD34 + CD38- AML blasts were treated with VEH or AOH (1 µM) for 24 h. **D** Effects of AOH on FAO (measured by ³H-palmitate levels, top) and OXPHOS (indicated by OCR levels, bottom), with the OCR comparison focused on maximal respiratory capacity. **E** Transmission electron microscopy (TEM) imaging of mitochondria. Enlarged images are shown. Scale bar, 1 μm. **F** Quantification of mitochondrial length (*n* = 30). Asterisks indicate statistically significant differences based on unpaired t-test analysis. **G-J** AOH effects on mitochondrial PCNA’s interaction with OPA1 and its impact on OPA1 stability in CD34 + CD38- AML blasts treated with VEH or AOH (1 µM) for 24 h. **G** Immunoblot analysis of mitofusion-regulated and mitochondrial metabolism-regulated proteins. **H** Mitochondrial fractions from treated cells were immunoprecipitated with anti-PCNA and immunoblotted with anti-OPA1 antibodies. Input loading controls are shown. **I** Cells were treated with cycloheximide, a translation inhibitor, (CHX, 10 µM), to assess protein stability, for indicated times, and lysates were immunoblotted with anti-OPA1 antibodies. **J** Left, lysates were immunoprecipitated with anti-OPA1 and immunoblotted with anti-MARCH5 antibodies. Input loading controls are shown. Right, ubiquitination assay with lysates immunoprecipitated with anti-OPA1 and immunoblotted with anti-Ub antibodies. **K-L** Antileukemic activities of AOH in vivo. Human primary AML blasts (1.0 × 10⁶) were injected intravenously into Es1(ko) SCID mice. After 7 days, mice were treated with vehicle control or AOH (100 mg/kg, BID, oral gavage, 3 weeks). **K** Leukemia burden at day 17 post-transplant, measured by the percentage of human CD45 + cells (left) and spleen size (right). **L** Left, effects of AOH treatment in vivo on FAO/OXPHOS in human CD45 + cells isolated from treated mice. FAO (top) and OXPHOS (bottom, indicated by OCR) levels were measured. Right, top Kaplan–Meier survival curve of primary transplanted leukemic mice treated with CON (black line, *n* = 10) and AOH-treated mice (red line, *n* = 10). Median survival (MS): 35 days (CON), 50 days (AOH). Right, bottom, Kaplan–Meier survival curve of secondary transplanted leukemic mice treated with CON (black line, *n* = 10) and AOH-treated mice (red line, *n* = 10). MS: 30 days (CON), 42 days (AOH). Statistical significance determined by Log-rank (Mantel–Cox) test (*p* < 0.0001)

AOH treatment, significantly inhibited proliferation, colony formation, and induced apoptosis at 24 h in a dose-dependent manner, in seven representative AML cell lines and primary CD34 + CD38- blasts (enriched for LSCs), while sparing normal CD34 + CD38- mononuclear cells (MNCs) [enriched for hematopoietic stem cells (HSCs)] (Fig. 1A-B and Sup. Figure S1). Untargeted metabolomic analysis identified 198 and 213 differentially abundant metabolites in AOH-treated (0.5 µM) primary CD34 + AML cells versus DMSO-treated or untreated controls (Adj. *p* < 0.05, Sup. Figure S2A and Sup. Table S1). To this end, AOH treatment led to reduced NAD+, FAD, and ATP levels, indicating decreased OXPHOS. Additionally, phospholipids, long-chain fatty acids, and acyl carnitines increased, while acetyl carnitine decreased, suggesting increased phospholipid synthesis and reduced FAO (Fig. 1C and Sup. Table S1) [10, 11]. Utilizing Seahorse and FAO functional assays, we confirmed that AOH treatment decreased FAO and OXPHOS (lower OCR) in CD34 + CD38- AML blasts (Fig. 1D and Sup. Figure S2B). Transmission electron microscope revealed AOH (1 µM) significantly reduced mitochondrial length (Fig. 1E-F), suggesting a potential inhibition of mitofusion. AOH treatment for 24 h also reduced levels of mitofusion-regulated proteins (e.g., OPA1, MFN1) and FAO/OXPHOS-regulated proteins (e.g., BCL-2, CPT1B, NRF2) in HL-60 cells and CD34 + CD38- AML blasts (Fig. 1G and Sup. Figure S3A). PCNA contains an AlkB homolog 2 PCNA-interacting motif (APIM), which binds to OPA1. Treatment with AOH disrupted the APIM -mediated OPA1- mitochondrial PCNA binding (Sup. Figure S3B-C), which led to increased OPA1 binding to its E3 ligase, MARCH5, and ubiquitination and accelerates degradation of this protein (Fig. 1H-J and Sup. Figure S3D). Thus, these results suggest that AOH inhibits mitofusion and mitochondrial oxidative metabolisms in LSC-enriched blast subpopulation by targeting cytoplasmic PCNA [5].

**Fig. 2:**
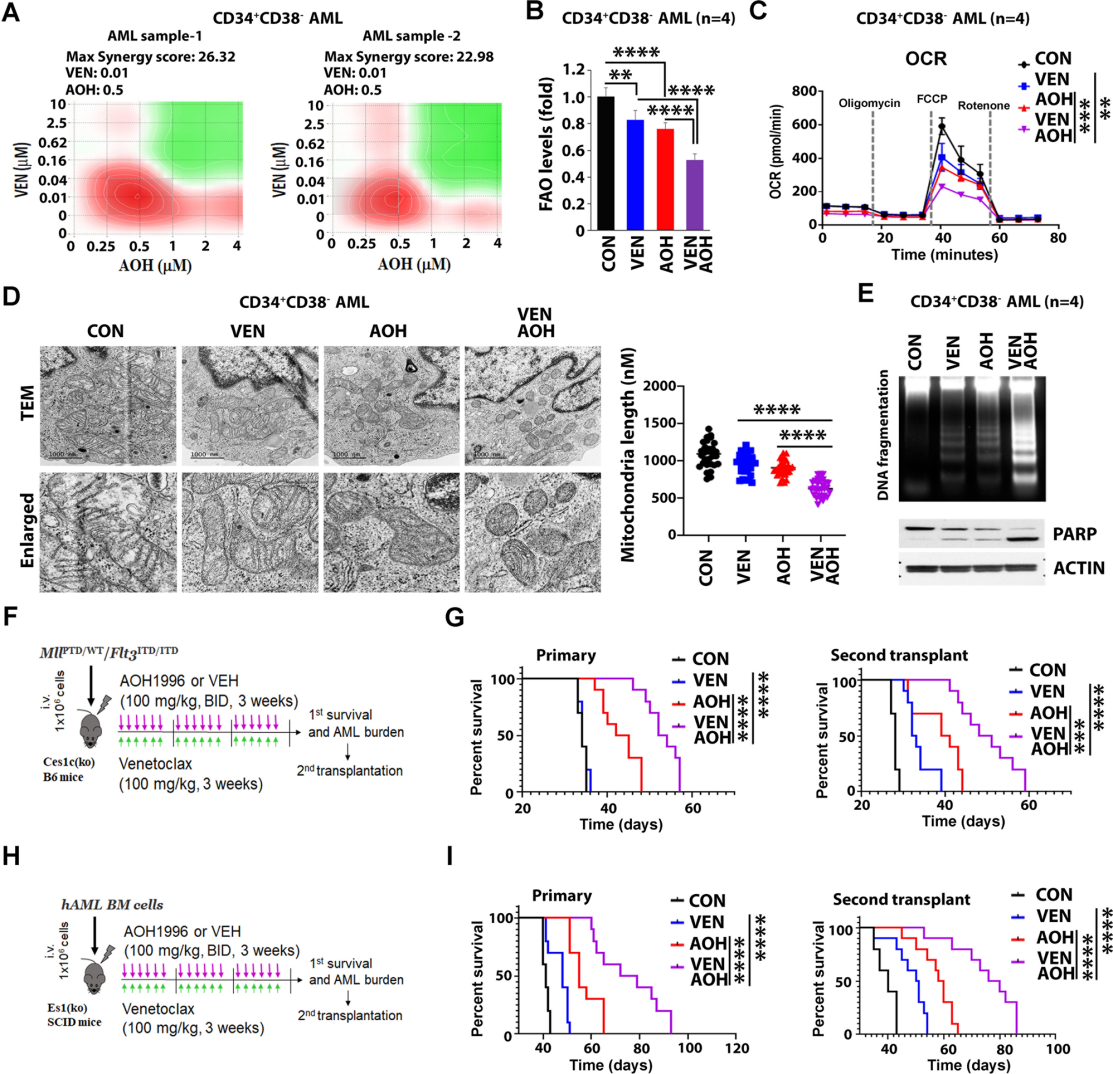
Synergistic effects of AOH and VEN in vitro and in vivo. **A** Synergistic effect of AOH and VEN on LSC-enriched AML blasts. Two primary CD34 + CD38- AML samples were used: AML sample-1 (corresponding to AML-2 in Table S2) and AML-sample 2 (corresponding to AML-3 in Table S2). Cells (1 × 10^5^ cells/mL, *n* = 3) were treated with indicated concentration of AOH and VEN. Levels of cell proliferation were evaluated and synergy score of the drug combination was calculated. Maximum synergy scores were 26.32 for AML sample-1 and 22.98 for AML sample-2. **B-D** Combinatorial effects of AOH and VEN on FAO/OXPHOS levels and mitochondrial length of LSC-enriched AML blasts. Primary CD34 + CD38- AML blasts (*n* = 4) were treated with DMSO (CON), AOH (1 µM), VEN (20 nM), or combination of AOH and VEN for 24 h. **B** FAO levels. **C** OXPHOS levels (indicated by OCR), with OCR comparisons focused on maximal respiratory capacity. **D** Mitochondria length. Left, represented TEM images. Right, quantification of mitochondria length (*n* = 30). **E** Primary CD34 + CD38- AML blasts (*n* = 4) were treated with DMSO (CON), VEN (20 nM), AOH (1 µM), or combination of AOH and VEN for 24 h. Top, DNA fragmentation. Bottom, PARP cleavage. ANOVA test was performed for multiple group comparisons prior to statistical analysis of each two group comparisons. **F-G** Combinatorial effects of AOH and VEN on Mll^PTD/WT^/Flt3^ITD/ITD^ AML mouse model. Kaplan-Meier curve employing log-rank test was used to find statistical significance. **F** Experimental design for AOH and VEN combined treatment. 1 × 10^6^ Mll^PTD/WT^/Flt3^ITD/ITD^ BM MNCs were intravenously injected into normal Ces1c(ko) B6 WT recipients. The transplanted mice were then randomly divided into 4 groups 7 days post-transplant (*n* = 10/group) and treated with either vehicle (CON), AOH (100 mg/kg, BID, PO, 21 days), VEN (100 mg/kg, daily, PO, 21 days) or AOH/VEN at the same doses of single agents. On day 28 post-transplant, 10^6^ BM MNCs cells from each treatment group were harvested for secondary transplant. **G** Left, Kaplan–Meier survival curve of primary transplanted leukemic mice treated with CON (black line, MS 34 days), VEN (blue line, MS 34 days), AOH (red line, MS 43.5 days), or AOH/VEN (purple line, MS 54 days). Right, Kaplan–Meier survival curve of secondary transplanted leukemic mice treated with CON (black line, MS 28 days), VEN (blue line, MS 32.5 days), AOH (red line, MS 41 days), or AOH/VEN (purple line, MS 49.5 days). **H-I** Combinatorial effects of AOH and VEN on FLT3-WT PDX AML model. **H** Experimental design for AOH and VEN combined treatment. hCD45 + BM FLT3-WT AML cells (1 × 10^6^ cells/mouse) were transplanted into Es1(ko) SCID mice to generate a cohort of AML bearing PDX mice. The transplanted mice were then randomly divided into 4 groups 7 days post-transplant (*n* = 10/group) and treated with either vehicle (CON), AOH (100 mg/kg, BID, PO, 21 days), VEN (100 mg/kg, daily, PO, 21 days) or AOH/VEN at the same doses of single agents. On day 28, 10^6^ BM MNCs cells from each treatment group were harvested for secondary transplant. **I** Left, Kaplan–Meier survival curve of primary transplanted leukemic mice treated with CON [black line, median survival (MS) 41 days], VEN (blue line, MS 48 days), AOH (red line, MS 55 days), or AOH/VEN (purple line, MS 75.5 days). Right, Kaplan–Meier survival curve of secondary transplanted leukemic mice treated with CON (black line, MS 40 days), VEN (blue line, MS 51 days), AOH (red line, MS 60 days), or AOH/VEN (purple line, MS 76 days)

In vivo, we demonstrated a significant antileukemic activity of AOH in AML PDXs. Compared to vehicle-treated mice, PDX mice receiving AOH (100 mg/kg, BID, 3 weeks) showed reduced leukemia burden (percentage of hCD45 + in peripheral blood and spleen size) and extended median survival (CON: 35 days vs. AOH: 50 days) (Fig. 1K-L). Analysis of human CD34 + cells from the bone marrow of treated mice, showed decreased FAO/OXPHOS levels (Fig. 1L, **left**). The antileukemic effect decreased LSC burden, as evidenced by the longer survival of recipients of BM MNCs from AOH-treated donors compared with recipients of BM MNCs from vehicle-treated donors in subsequent transplants (CON: 30 days vs. AOH: 42 days) (Fig. 1L, **right bottom**). Of note, AOH in combination with a BCL-2 inhibitor, venetoclax (VEN), resulted in a synergistic activity in vitro, as shown by inhibition of FAO/OXPHOS, reduction in mitochondria length, and increased apoptosis of CD34 + CD38- AML blasts (Fig. 2A-E). The AOH/VEN combination significantly extended survival in both primary and secondary transplant experiments in murine (Mll^PTD/WT^/Flt3^ITD/ITD^) [12] models compared to either agent alone. Primary survival (days): CON: 34, VEN: 34, AOH: 43.5, AOH/VEN: 54; secondary survival (days): CON: 28, VEN: 32.5, AOH: 41, AOH/VEN: 49.5 (Fig. 2F-G and Sup. Figure S4A-B). Similar results were seen in PDX AML models (Fig. 2H-I). Notably, the drug treatment did not affect mouse weight (Sup. Figure S4C).

In summary, AOH exhibits potent antileukemic activity in AML models by inhibiting mitochondrial PCNA-regulated dynamics and metabolism and reducing LSC burden. Of note, we also observed enhanced activity when AOH is combined with VEN. While VEN is FDA-approved for AML, AOH is a novel compound currently in clinical trials for solid tumors and undergoing IND-enabling studies for leukemia.

## Electronic supplementary material

Below is the link to the electronic supplementary material.

Supplementary Material 1

Supplementary Material 2

Supplementary Material 3

Supplementary Material 4

Supplementary Material 5

Supplementary Material 6

## Data Availability

No datasets were generated or analysed during the current study.
