# Correction: AOH1996 targets mitochondrial dynamics and metabolism in leukemic stem cells via mitochondrial PCNA inhibition

**DOI:** 10.1186/s40164-025-00650-7

**Published:** 2025-04-10

**Authors:** HyunJun Kang, Melissa Valerio, Jia Feng, Long Gu, Dinh Hoa Hoang, Amanda Blackmon, Shawn Sharkas, Khyatiben Pathak, Jennifer Jossart, Zhuo Li, Hongyu Zhang, Bin Zhang, Patrick Pirrotte, J. Jefferson P. Perry, Robert J. Hickey, Linda Malkas, Guido Marcucci, Le Xuan Truong Nguyen

**Affiliations:** 1 Department of Hematologic Malignancies Translational Science, Beckman Research Institute and City of Hope National Medical Center, Duarte, CA USA; 2 Department of Hematology, Peking University Shenzhen Hospital, Shenzhen, China; 3 Department of Molecular Diagnostics & Experimental Therapeutics, Beckman Research Institute of City of Hope, Duarte, CA USA; 4 Early Detection and Prevention Division, Translational Genomics Research Institute, Phoenix, AZ USA; 5 Beckman Research Institute, City of Hope National Medical Center, Duarte, CA USA; 6 Department of Molecular Medicine, City of Hope National Medical Center, Duarte, CA USA; 7 City of Hope, Duarte, CA 91010 USA

**Correction: Experimental Hematology & Oncology (2024) 13:123** 10.1186/s40164-024-00586-4

Following the publication of this article [1], the authors identified errors in the funding and competing interests sections. Specifically,

1. The IDDV program funding was mistakenly included and should be removed from the funding and acknowledgements section.
2. LG, JPP, RJH, and LM should be declared as shareholders of RLL Inc.

The revised sections are as follows:

Funding

This study was partially supported by the National Cancer Institute of the National Institutes of Health under award number P30CA33572.

Competing interests

LG, JPP, RJH and LM are share holders of RLL Inc. The remaining authors declare that there is no conflict of interest regarding the publication of this article.

Acknowledgements

We would like to thank the Animal Resources Center and the various core facilities—including Analytical Cytometry, Light Microscopy, Electron Microscopy, and Integrated Mass Spectrometry Shared Resource Cores—at the City of Hope Comprehensive Cancer Center for their invaluable support. This support is made possible by the National Cancer Institute of the National Institutes of Health under award number P30CA33572. Our heartfelt appreciation also goes out to the City of Hope Comprehensive Cancer Center, the patients, and their physicians for providing the essential materials for this study.

The original article has been corrected.
