# Supplementary material for: AOH1996 targets mitochondrial dynamics and metabolism in leukemic stem cells via mitochondrial PCNA inhibition
Source: Exp Hematol Oncol. 2024 Dec 28;13:123. doi: 10.1186/s40164-024-00586-4 (PMC11681632; doi:10.1186/s40164-024-00586-4)
Supplement: Supplementary file 1 — Supplementary Material 1 [file 40164_2024_586_MOESM1_ESM.docx]

**SUPPLEMENTAL INFORMATION**

1. **Supplementary Materials and Methods** (Page 2 to Page 10)
2. **Supplementary Reference** (Page 10)
3. **Supplementary Tables:** (Page 11 to Page 12)

**+ Table S1.** Differential abundance of metabolites in primary CD34+ AML blasts treated with AOH compared to DMSO or non-treat control (attached Excel file)

**+ Table S2.** Characteristics of patient samples

**+ Table S3.** List of antibodies used for IP, IB, and IF analysis

1. **Supplementary Figures and Tables Legends** (Page 13 to Page 15)

+ **Figure S1.** Effects of AOH1996 (AOH) on Proliferation and Apoptosis of AML Cell Lines and Primary AML Blasts

+ **Figure S2.** Effects of AOH1996 (AOH) on the metabolic profile of leukemic stem cells (LSCs)

+ **Figure S3.** Effects of AOH1996 (AOH) on mitochondrial PCNA binding with OPA1

+ **Figure S4.** Synergistic effect of AOH1996 (AOH) and venetoclax (VEN) *in vivo*

*+* **Table S1.** Differential abundance of metabolites in primary CD34+ AML blasts treated with AOH compared to DMSO or non-treat control

+ **Table S2.** Characteristics of patient samples.

+ **Table S3.** List of antibodies used for IP, IB, and IF analysis.

1. **Supplementary Figures**

**Supplementary Materials and Methods**

**Human samples**

Normal hematopoietic stem cells (HSCs) and acute myeloid leukemia (AML) samples were obtained from healthy donors and patients at City of Hope National Medical Center (COHNMC) under Institutional Review Board-approved protocols (#06229, #03162, #07047, #18067). Primary AML cells were cultured in RPMI (Roswell Park Memorial Institute) medium supplemented with 20% fetal bovine serum (FBS), 100 units/mL of penicillin/streptomycin, and 10 ng/mL of hIL-3. The cells were maintained at 37°C in an environment with 5% CO2 and high humidity. These protocols follow the guidelines of the Department of Health and Human Services and adhere to the Declaration of Helsinki. Written informed consent was obtained from donors (#06229) and patients (#03162, #07047, #18067) prior to sample collection. Detailed patient characteristics for primary AML samples are provided in **Sup. Table S2**.

**Isolation of mononuclear cells from patient samples**

Each patient specimen was initially transferred into a 50 mL conical tube and diluted to 25 mL with warm 1x Dulbecco’s phosphate-buffered saline (DPBS) containing 2% fetal bovine serum (FBS). The specimen was then gently layered over 20 mL of Ficoll-Paque Plus in another 50 mL conical tube and centrifuged at 300 g for 32 minutes without braking. The peripheral blood mononuclear cell (PBMC) and plasma layer was carefully collected into a fresh 50 mL tube and diluted to 50 mL with warm 1x DPBS. This was followed by a second centrifugation at 2400 rpm for 8 minutes. After discarding the supernatant, the cell pellet was resuspended in 10 mL of warm 1x DPBS, cell count and viability were measured, and the sample was frozen for later analysis.

**Design and synthesis of AOH1996**

AOH1996, which targets a cancer-associated isoform of PCNA, was discovered, characterized, and synthesized, as previously described^1^.

**Cell cultures and Chemicals**

Kasumi-1, U937, THP-1, Molm13, HL-60, KG-1a, and MV-4-11 cells were obtained from the American Type Culture Collection (ATCC) and cultured in either IMDM (Iscove’s Modified Dulbecco’s Medium) or RPMI (Roswell Park Memorial Institute) medium supplemented with 10% fetal bovine serum (FBS) and 100 units/mL of penicillin/streptomycin. The cells were maintained at 37°C in an environment with 5% CO2 and high humidity. Human cell lines sourced from ATCC more than 6 months prior to manuscript submission, or those not cryopreserved at an early passage, were authenticated using ATCC’s human short tandem repeat (STR) DNA profiling service. Cell line morphology was regularly monitored, and mycoplasma testing was routinely performed using a mycoplasma detection kit (Roche, Germany). Venetoclax, cycloheximide (CHX), and MG132 were purchased from Selleckchem (Houston, TX). An oral dosing solution was prepared by dissolving AOH1996 (40 mg) in a mixture of Kolliphor EL (840 mg, Sigma C5135) and Poloxamer P124 (120 mg, Spectrum Chemical P1168).

**Cell proliferation assay**

To assess the effect of AOH1996 on AML cell proliferation, a WST-1 (water-soluble tetrazolium salt) assay (Cat #5015944001, Millipore Sigma) was performed. Cells were plated at a density of 100,000 cells per well in a 96-well plate and treated with varying concentrations of AOH1996 for 24 hours at 37°C. After treatment, 10 µL of WST-1 solution was added to 100 µL of culture medium, and the plate was incubated for an additional 2 to 3 hours at 37°C. Formazan, the product of WST-1 metabolism, was measured using a multi-well spectrophotometer at 450 nm. The synergistic effect of AOH1996 and venetoclax (ABT-199, Cat # S8048, Selleckchem) against primary AML cells was evaluated with various combinations of doses (AOH: 0.25, 0.5, 1.0, 2.0, 4 µM; ABT-199: 0.002, 0.01, 0.04, 0.16, 0.62, 2.5, 10 µM).

**Annexin-V staining**

Apoptosis was evaluated by flow cytometry using Annexin-V and DAPI double staining. Cells were collected, washed twice with Annexin-V binding buffer (BD Biosciences), and resuspended in 100 μL of buffer containing Annexin-V APC (BD Biosciences). After a 15-minute incubation in the dark at room temperature, cells were washed again and resuspended in 300 μL of buffer. DAPI (Sigma-Aldrich) was added immediately before analysis on an LSR II flow cytometer (BD Biosciences). Cells positive for Annexin-V and negative for DAPI (Annexin-V+/DAPI-) were classified as apoptotic.

**Colony forming assay**

The potential of leukemic stem cells (LSCs) and hematopoietic stem cells (HSCs) was assessed using a colony formation assay. For this, 5 × 10³ cells treated with AOH1996 (0.5 µM) or DMSO were mixed with 1.5 mL of H4434 MethoCult (Stem Cell Technologies) and thoroughly vortexed. The cell mixture was then plated in a 6-well plate and incubated at 37°C. Colony formation was evaluated between days 12 and 14 of culture.

**Metabolomic analysis**

To ensure sufficient material for accurate analysis, untreated CD34+ primary AML blasts, including the LSC fraction, as well as those treated with DMSO or AOH1996 (0.5 µM), were selected for metabolomic analysis. To minimize potential confounding effects on cell viability, cells were treated with low dose of AOH1996 (0.5 µM), and only live cells were included in the analysis. Metabolomic analysis was conducted as previously described^2^. In brief, 3×10^6 cells were subjected to metabolite extraction using a methanol: acetonitrile: water mixture (2:1:1, v/v/v) spiked with four internal standards (d_8_-Valine, ^13^C_3_-Phenylalanine, ^13^C_6_-adipic acid, d_4-_succinic acid). The cell lysates underwent vortexing and three freeze-thaw cycles, followed by centrifugation at 15,000 rpm for 10 minutes at 4°C, yielding two aliquots. One was analyzed by HILIC LC-MS, and the other was vacuum-dried and reconstituted in 80% water, 20% acetonitrile for RP LC-MS analysis. System suitability was determined using in house plasma metabolite standard, and an aliquot of each sample was pooled to prepare pooled QC samples, which were used for monitoring batch performance and normalization. Data were acquired on an Ultimate 3000 RSLC with HPG pump coupled to an Orbitrap Fusion Lumos Tribrid mass spectrometer (ThermoFisher) in both HILIC and RP modes, using positive and negative ionization. MS1 data were collected over a 70-1500 *m/z* mass range, and MS/MS data were acquired using stepped HCD collision energy. Raw data were analyzed on Compound Discoverer 3.2 (ThermoFisher) using KEGG, HMDB, mzCloud and lipid MAPS databases for metabolite annotation and relative quantitation. Metabolite extract from these samples were also subjected to semi-targeted analysis focusing on 17 metabolites (Glucose, glucose-6 phosphate, pyruvate, lactate, acetyl CoA, fumarate, malate, succinate, citrate, NAD, FAD, NADH, ADP, ATP, carnitine, acetyl carnitine and palmitoyl carnitine). The analysis was performed on Vanquish UPLC coupled to TSQ-Altis triple quadrupole mass spectrometer (ThermoScientific) using Hypercarb column chromatography as described previously^3^ and multiple reaction monitoring (MRM) assay. The assay used synthetic standards of above listed metabolites (Sigma Aldrich, MO) for MRM and retention time match. The raw data were processed using Quan browser (Thermofisher) for retention time match and peak integration. Both untargeted and targeted data were processed using Variance Stabilizing Normalization (VSN). An Analysis of variance (ANOVA) was performed to determine differentially abundant metabolites between the three groups, AOH, DMSO, and Non-treat (NT), followed by a Tukey post hoc to examine specific pairwise comparisons. Metabolites with Adj. p < 0.05 considered to be significantly different. Only annotated endogenous metabolites were used for data representation, and statistical analysis was performed using a two-tailed t-test and ANOVA, focusing on annotated non-redundant endogenous metabolites. The data for differentially abundant metabolites are provided in **Sup. Table S1**.

**FAO assay**

Live cells were sorted for the FAO assay to avoid confounding effects from cell death. Cells were washed with HBSS and incubated with 200 µL of a mixture containing [3H]-palmitic acid (1 mCi/mL, Perkin Elmer) bound to fatty acid-free albumin (100 µM, at a palmitate-to-albumin ratio of 2:1) and 1 mM l-carnitine at 37°C for 2 hours. After incubation, the supernatant was mixed with 200 µL of chilled 10% trichloroacetic acid and centrifuged at 3000 × g for 10 minutes at 4°C. A 350 µL aliquot of the supernatant was neutralized with 55 µL of 6 N NaOH, then passed through an ion exchange column with Dowex 1X2 resin (Sigma Aldrich). The radioactive fraction was eluted with water, and radiation levels were measured by liquid scintillation counting.

**Seahorse assay**

Live cells were sorted for the Seahorse assay to avoid confounding effects from cell death. Each well of a Seahorse XF-96 cell culture microplate was seeded with 300,000 cells in 180 µL of culture medium and incubated overnight at 37 °C with 5% CO2. For controls, three wells were left empty and filled with Seahorse media, which included basal XF media, 5.5 mM glucose, 1 mM sodium pyruvate, and 4 mM glutamine (pH 7.4). A Seahorse sensor cartridge was preconditioned 12 hours before the assay by soaking in Seahorse Calibrant solution per the manufacturer’s instructions in a CO2-free incubator at 37 °C. On the assay day, cells were washed and replaced with Seahorse media. The sensor cartridge was mounted on the plate and incubated in a CO2-free environment at 37°C for 60 minutes. The Seahorse XF96 Analyzer (Agilent, Santa Clara, CA) was then used to perform the assay, with sequential injections of oligomycin (1.5 µM), FCCP (1 µM), and Rotenone/Antimycin A (0.5 µM) following the Cell Mito Stress Test protocol.

**Transmission Electron Microscope (TEM)**

Cells treated with AOH1996 or DMSO were fixed in 2.5% glutaraldehyde in 0.1M Cacodylate buffer (Na(CH3)2AsO2·3H2O), pH 7.2, at 4°C. Standard transmission electron microscopy (TEM) preparation protocols were followed, including post-fixation with osmium tetroxide, serial ethanol dehydration, and embedding in Eponate. Ultra-thin sections (70 nm) were prepared using ultramicrotomy, post-stained, and imaged with a FEI Tecnai 12 TEM equipped with a Gatan OneView CMOS camera at a nominal magnification of 11,000x.

**Immunoblotting analysis**

Cells were washed and harvested in ice-cold PBS, then lysed in RIPA buffer containing 10 mM protease inhibitor cocktail (Thermo Scientific). For immunoblotting, 50 µg of each lysate was separated on NuPAGE 4-12% gradient gels (Invitrogen), and the resulting immunocomplexes were visualized using enhanced chemiluminescence reagent (Thermo Scientific). For protein stability studies, cells were treated with the translation inhibitor cycloheximide (CHX, 10 μM) to monitor time-dependent protein degradation. Details of the antibodies used are provided in **Sup. Table S3.**

**Ubiquitin assay**

Cells treated with AOH1996 or DMSO were collected and lysed in RIPA buffer containing 10 mM protease inhibitor cocktail (Thermo Scientific). Before AOH1996 or DMSO treatment, cells were pre-treated with the protease inhibitor MG132 (10 μM) to prevent protein degradation, enabling accurate assessment of protein ubiquitination levels. After lysis, 500 µg of the cell lysate was incubated overnight at 4°C with specific antibodies. Protein A/G agarose beads (50 µL, Santa Cruz) were then added, and the mixture was gently inverted for 3 hours at 4°C. The immunoprecipitated complexes were separated using NuPAGE 4-12% gradient gels (Invitrogen) and analyzed by immunoblotting with an anti-Ubiquitin antibody.

**Mice**

All transplants were performed through intravenous (i.v.) injection via the tail vein. Human primary FLT3 wild-type AML blasts^4-6^ were injected into sublethally irradiated (2.0 Gy, XRAD 320-Precision X-Ray) 6-8-week-old Es1(ko) SCID mice. Mll^PTD/WT^/Flt3^ITD/ITD^ knock-in AML cells were injected into sublethally irradiated (4.5 Gy, XRAD 320-Precision X-Ray) 6-8-week-old Ces1c(ko) B6 mice. In vivo treatments, including vehicle (VEH), AOH1996 (AOH), or venetoclax (VEN), were administered via oral gavage beginning 7 days post-transplant for 21 days BID. 17 days post-transplant, peripheral blood was collected to determine leukemic engraftment via Flow Cytometric analysis. Upon treatment completion, mice were humanely euthanized, and spleen and bone marrow were collected. BM MNCs were flushed from femurs and tibias and analyzed via Seahorse Assays. The in vivo AOH dosage was determined based on previous pharmacokinetic, toxicology, and solid tumor studies^1^. AOH (40 mg) was dissolved in a mixture of Kolliphor EL (840 mg) and Poloxamer P124 (120 mg) to achieve a dosing solution concentration of 4% by weight. All mice were housed in an AAALAC-accredited facility, and experimental procedures complied with federal and state regulations, as well as institutional protocols approved by the City of Hope Institutional Animal Care and Use Committee.

**Cell isolation and flow cytometry**

Bone marrow mononuclear cells were extracted from femurs, tibias, and pelvis using a mortar and pestle. Murine peripheral blood was collected via retro-orbital bleeding. RBCs were lysed with ACK Lysis Buffer (Gibco Cat# A1049201). For fluorescence-activated cell sorting (FACS) analysis, cells were incubated with fluorescently labeled antibodies in PBS with 0.5% BSA for 15 minutes at 4°C. Flow cytometry was performed with a 5-laser BD LSRFortessa™ X-20 cell analyzer. Antibodies, such as mCD45, hCD33 and hCD45, were sourced from BioLegend. To isolate human primary blast/hematopoietic stem and progenitor cells (hHSPCs) with the CD34+CD38- immunophenotype, cells from Ficoll-separated bone marrow or cord blood were processed using the Human CD34+CD38- Cell Isolation Kit (Cat# 130-114-822, Miltenyi Biotec). CD34+ cells were first labeled with CD34-microbeads and separated by magnetic separation, followed by enrichment through CD38+ cell depletion. Data analysis was conducted using FlowJo software version 10.6.1.

For AML samples with a low blast percentage (less than 20%), CD123+ was used to enrich AML blasts through flow cytometry-based sorting^7^.

**DNA fragmentation assay**

Cells treated with AOH1996 or DMSO were lysed on ice for 60 minutes in 500 μL of lysis buffer containing 0.02% SDS, 1% Nonidet P-40, and 0.2 mg/mL proteinase K in PBS. Genomic DNA was extracted using the phenol/chloroform method, and the resulting pellet was dissolved in 50 μL of TE buffer with 10 mg/mL RNase, followed by a 2-hour incubation at 37°C. Afterward, 10 μg of DNA was loaded onto a 2% agarose gel and visualized under UV light.

**Immunocytochemistry**

Cells were collected, washed with ice-cold PBS, and then placed on glass slides using a Cytocentrifuge (CytoSpin4, 600 rpm, 10 minutes). The cells were subsequently rinsed with PBS, fixed in 4% paraformaldehyde for 15 minutes, and permeabilized with 0.5% Triton X-100 for 15 minutes. To block nonspecific binding, cells were incubated with 5% bovine serum albumin (BSA) for 30 minutes. Details of the primary antibodies used are listed in Supplementary Table S3. Secondary antibodies (anti-mouse/rabbit/goat-Alexa 594/488/647) were sourced from Thermo Scientific. Cell imaging was carried out using a Zeiss confocal laser-scanning microscope (Zeiss LSM 800), and nuclei were counterstained with ProLong Gold Antifade with DAPI (Molecular Probes, Invitrogen).

**Statistical analysis**

To compare means between two groups, data were analyzed using an unpaired, two-tailed Student’s t-test. Results from at least two independent experiments with triplicate measurements were included, unless specified otherwise. Mice survival was analyzed using Kaplan-Meier curves with the Log-rank test. For comparisons involving more than two groups, an ANOVA test was initially performed to identify statistically significant differences among groups. Depending on variance equality, confirmed by the F test, either the Student’s t-test or Welch’s t-test was then applied as a post hoc analysis for specific group comparisons. All statistical analyses were conducted using SigmaPlot 12.5 (Systat Software, Chicago, Illinois) and R v4.4.2 (https://www.R-project.org/), with two-sided tests. Data are presented as mean ± standard error (S.E.). Statistical significance was defined as p<0.05, with notations as follows: ns = not significant, * = p≤0.05, ** = p≤0.01, *** = p≤0.001, **** = p≤0.0001.

**Supplementary Reference**

1. Gu L, Li M, Li CM, et al. Small molecule targeting of transcription-replication conflict for selective chemotherapy. *Cell Chem Biol*. 2023;30(10):1235-1247.e1236.

2. Zhang B, Zhao D, Chen F, et al. Acquired miR-142 deficit in leukemic stem cells suffices to drive chronic myeloid leukemia into blast crisis. *Nature Communications*. 2023;14(1):5325.

3. Wang H, He X, Zhang L, et al. Disruption of dNTP homeostasis by ribonucleotide reductase hyperactivation overcomes AML differentiation blockade. *Blood*. 2022;139(26):3752-3770.

4. Kang H, Hoang DH, Valerio M, et al. OST-01, a natural product from Baccharis coridifolia, targets c-Myc-dependent ribogenesis in acute myeloid leukemia. *Leukemia*. 2024;38(3):657-662.

5. Wang H, Zhao D, Nguyen LX, et al. Targeting cell membrane HDM2: A novel therapeutic approach for acute myeloid leukemia. *Leukemia*. 2020;34(1):75-86.

6. Zhang Y, Park M, Ghoda LY, et al. IL1RAP-specific T cell engager depletes acute myeloid leukemia stem cells. *J Hematol Oncol*. 2024;17(1):67.

7. Gill S, Tasian SK, Ruella M, et al. Preclinical targeting of human acute myeloid leukemia and myeloablation using chimeric antigen receptor-modified T cells. *Blood*. 2014;123(15):2343-2354.

**Supplementary Table S2. Characteristics of patient samples**

| **Sample ID** | **Sample Type** | **Disease Status** | **Cytogenetic** | **Mutations** | **WBC (10^3^/μl)** | **PB Blasts (%)** | **BM Blasts (%)** | **Figure Reference per sample** |
| --- | --- | --- | --- | --- | --- | --- | --- | --- |
| AML-1 | PB | New Diagnosis | Normal | IDH2 Pos | 1.4 | 5 | >90 | S1B, S1F, S1G, 1A-B, 2B-E |
| AML-2 | BM | New Diagnosis | Normal | NPM1 Pos | 33 | 87 | 90 | S1B, S1F, S1G, 1A-F, S2, 2A-E |
| AML-3 | PB | Relapsed | Complex | FLT3-ITD Pos, NPM1 Pos | 46.4 | 66 | >90 | S1B, S1F, S1G, 1A-B, 1D-F, 2A-E |
| AML-4 | PB | New Diagnosis | Inv(16) | FLT3-ITD Neg |  | 67 | 90 | S1F, 1A, 1D-F, S2C, 2B-E |
| AML-5 | BM | New Diagnosis | Normal | CEBPA Pos | 33 | 70 | 60 | 1G-J, S3D |
| AML-6 | PB | Relapsed | Normal | FLT3-ITD Neg, NPM1 Neg, IDH1 Neg | 29 | 91 | >90 | 1G-J |
| AML-7 | BM | Refractory | Complex | TET2 Pos | 105 | 34 | 15 | 1G-J |
| AML-8 | PB | Relapsed | t(16;16), trisomy 21, trisomy 22 | FLT3-ITD Neg |  | 94 | 67 | 1G-J |
| AML-9 | BM |  | Normal | FLT3-ITD Neg |  | 59 | 90 | 1K-L, 2H-I |

**Supplementary Table S3. List of antibodies used for IP, IB, and IF analysis**

| No | Antibody name | Information |
| --- | --- | --- |
| 1 | Anti-PARP antibody | Cat# 9542, Cell Signaling |
| 2 | Anti-BCL-2 antibody | Cat# sc-7382, Santa Cruz |
| 3 | Anti-OPA1 antibody | Cat# 80471, Cell Signaling |
| 4 | Anti-MARCH5 antibody | Cat# PA5-25584, Thermo Fisher |
| 5 | Anti-ACTIN antibody | Cat# sc-47778, Santa Cruz |
| 6 | Anti-NRF2 antibody | Cat# ab62352, Abcam |
| 7 | Anti-CPT1B antibody | Cat# ab134988, Abcam |
| 9 | Anti-MFN1 antibody | Cat# 14739, Cell Signaling |
| 10 | Anti-Ub antibody | Cat# 07-357, Millipore |
| 11 | Anti-PCNA antibody | Cat# sc-53408, Santa Cruz |
| 12 | Anti-TOM20 antibody | Cat# sc-136211, Santa Cruz |

**Supplementary Figure and Table Legends**

**Figure S1.** **Effects of AOH1996 (AOH) on proliferation and apoptosis of AML cell lines and primary AML blasts. A-B** Effect of AOH on AML cell viability. AML cell lines Kasumi-1, U937, THP-1, Molm13, HL-60, KG-1a, and MV4-11 (**A**) and primary AML blasts (**B**) (10^5^ cells) were incubated with various doses of AOH for 24 hours. Cell viability was assessed using the WST-1 proliferation assay. IC50 values: Kasumi-1, 0.992 µM; U937, 0.358 µM; THP-1, 3.838 µM; Molm13, 0.495 µM; HL-60, 0.921 µM; KG-1A, 0.899 µM; MV-4-11, 0.913 µM; AML-1, 0.864 µM; AML-2, 1.562 µM; AML-3, 0.394 µM. **C-E** Effect of AOH on proliferation and apoptosis. AML cell lines Kasumi-1, U937, THP-1, Molm13, HL-60, KG-1a, and MV4-11 (10^5^ cells) were treated with 1 µM AOH for 24 hours. **C** Cell proliferation was measured by WST-1 assay. **D** Apoptosis levels were assessed using annexin V staining and flow cytometry. **E** DNA fragmentation and PARP cleavage were analyzed by agarose gel electrophoresis and Western blot, respectively. Data are presented as mean ± SE from two independent experiments with triplicate determinations. Statistically significant differences are indicated by asterisks based on unpaired t-test analysis. **F** Effect of AOH (1 µM) on apoptosis of LSC-enriched AML blasts. CD34+CD38- cells isolated from primary MNCs (n = 4) or AML blasts (n = 4) were treated with AOH. Top panel shows DNA fragmentation; bottom panel shows PARP cleavage levels. **G** Effect of AOH on colony formation of LSC-enriched AML blasts. CD34+CD38- AML blasts (left) or MNCs (right) (2 × 10^5^ cells/mL, n = 3) were treated with DMSO (control) or indicated doses of AOH for 24 hours, then plated in methylcellulose. After 14 days, colonies were imaged and counted using a light microscope. Representative colony images are shown.

**Figure S2. Effects of AOH1996 (AOH) on the metabolic profile of leukemic stem cells (LSCs). A** Unsupervised hierarchical clustering reveals significant changes in metabolite abundances in primary CD34+ AML blasts treated with DMSO control (VEH) or AOH (0.5 µM) for 24 hours (n=4 per group), analyzed through untargeted metabolomics. **B** Glycolysis (indicated by ECAR) was measured by Seahorse assay in primary CD34+CD38- AML blasts treated with VEH or AOH (1 µM) for 24 hours.

**Figure S3. Effects of AOH1996 (AOH) on mitochondrial PCNA binding with OPA1. A** HL-60 cells were treated with either DMSO or various concentrations of AOH for 24 hours. Cell lysates were then immunoblotted with the indicated antibodies. **B** and **C** Effects of AOH on mitochondrial PCNA-OPA1 binding. **B** Left, co-crystal structure of the ZRANB3 APIM motif peptide bound to PCNA superimposed onto the PCNA co-complex. Right, the AOH1996-1LE compound within the APIM-motif binding pocket potentially blocks OPA1 peptide binding. **C** The AOH1996-1LE compound binds to PCNA, causing steric clashes with residues V1282 and Lys1280. This binding modifies the APIM motif binding pocket, potentially disrupting interaction with the OPA1 peptide. Conformational changes in the interdomain-connector loop (Gln125-Ile128) and the loop formed by Ala231-Pro234 may further impair binding between OPA1 and PCNA. **D** Effects of AOH on OPA1 expression in mitochondria. Primary CD34+CD38- AML blasts were treated with either DMSO or AOH (1 µM) for 24 hours. Cells were stained with anti-TOM20 (green) and anti-OPA1 (red) antibodies, and images were captured using a confocal microscope. Scale bar, 10 µm.

**Figure S4. Combinatorial effect of AOH1996 (AOH) and venetoclax (VEN) *in vivo***. 1×10^6^ Mll^PTD/WT^/Flt3^ITD/ITD^ BM MNCs were intravenously injected into normal Ces1c(ko) B6 WT recipients. The transplanted mice were then randomly divided into 4 groups (n = 10/group) and treated with either vehicle (CON), AOH (100 mg/kg, BID, PO, 21 days), VEN (100 mg/kg, daily, PO, 21 days) or AOH/VEN at the same doses of single agents. On day 21, 10^6^ BM MNCs cells from each treatment group were harvested for secondary transplant. **A** Left, WBC (x10^3^) in the bone marrow (BM) of primary transplant mice. Right, representative image of the spleen. **B** WBC (x10^3^) in the peripheral blood (PB) of secondary transplant mice. **C** Graphs illustrating no changes in body weight for B6 (left) and SCID (right) mice during 21 days of treatment with vehicle, AOH, VEN, or the AOH/VEN combination.

**Table S1.** **Differential abundance of metabolites in primary CD34+ AML blasts treated with AOH compared to DMSO or non-treat control.** **A** Untargeted metabolomic profiling of primary CD34+ AML blasts treated with vehicle (DM), untreated (NT), or AOH (0.5 μM) for 24 hours. Comparisons include normalized area across conditions, fold changes, p-values, and statistical tests performed. **B** Targeted analysis of metabolites from primary CD34+ AML blasts treated with vehicle (DM), nontreated (NT) or AOH (0.5 μM) for 24 hours.

**Table S2. Characteristics of patient samples.**

**Table S3. List of antibodies used for IP, IB, and IF analysis.**
